# Outdoor domestic cats and wildlife: How to overrate and misinterpret field data

**DOI:** 10.3389/fvets.2022.1087907

**Published:** 2022-12-14

**Authors:** Dennis C. Turner

**Affiliations:** ^1^Institute for Applied Ethology and Animal Psychology, I.E.A.P./I.E.T., Horgen, Switzerland; ^2^Vetsuisse Faculty, University of Zurich, Zurich, Switzerland

**Keywords:** wildlife, predation, biodiversity, cats, felis catus

## 1. Introduction

Lynn et al. (1) have questioned the moral panic over outdoor domestic cats destroying wildlife and reducing biodiversity. Although some early studies in Australia drew attention to the problem of free-ranging outdoor cats, two other recent studies about cat predation have attracted broader international media attention (2, 3) and have become the focus of considerable controversy. The current author attempts a fair appraisal of such studies and their conclusions, or rather of the interpretation and conclusions drawn by others at the expense of outdoor cats. In this review, I explain why the results of published studies purporting to show that cats are a main culprit for the disappearance of endemic wildlife on the species level, on the continents as opposed to small oceanic islands, should be questioned. This will indicate the information we still need, and need to integrate, before drawing any conclusions that condemn or exonerate free- roaming cats, in order to examine correctly the role that outdoor cats actually play in relation to wildlife.

Quite often domestic cats are considered by conservationists to be an invasive species. The cat itself is mostly responsible for its domestication (“self- domestication,” albeit with some help from ancient peoples) and the expansion of its geographic range from the Fertile Crescent area to the East, North and South. The domestic cat is an extremely flexible, adaptable species and a successful predator, in most cases capable of surviving without human support (4, 5). But as Ottoni et al. (6) have shown the domestic cat's dispersal gained momentum during the Classical period, when the Egyptian cat successfully spread throughout the Old World.

Further, people arguing against cats usually assume one of two vantage points: either that of (prey) animal protection and welfare (“the poor prey animals”), or that of prey species/biodiversity conservation. This essay critically addresses only the second vantage point.

## 2. What is known about domestic cat predatory behavior and predation?

Various facts are available from field studies throughout the world and need to be considered in any study examining the effect of cats on wildlife. A review of all published studies (over 60) on various aspects of cat predatory behavior in 1986 by Fitzgerald (7) brought many facts to light which should be considered in any estimate of the impact of cats on their prey populations.

Many of those studies were based on *prey carried home by the cats* and collected by the owners for the researchers. However, it has been shown that there are differences both in the numbers and species between prey carried home and prey consumed in the field (8, 9), also implying that any estimates of the numbers of prey killed by cats may be higher. Further, it is known that there are differences between the species of prey killed and consumed and those abandoned [e.g., (8, 10, 11)].

Gut analyses of road killed cats and cats shot in different habitats also yield information that needs to be considered. These have indicated that most mammalian prey are from those species living on fields/meadows or (at most) forest edges; male cats examined from forest areas rarely have prey species in their digestive tracts that live in the forest, but rather field prey species or “human prepared foods” (12, 13). Further, some studies report on prey consumed based on percent volume in the gut, while others use estimated percent occurrence of the different prey types (7). These differences should be taken into account when combining the data from different studies to assess impact on prey types, but that is rarely done.

Both the habitat type (fields/meadows or forest) and general housing density (rural, suburban, urban) where a study was/is conducted should also be considered. What one sees in urban or suburban areas is not necessarily representative or problematic. Some potential prey species (e.g., house sparrows, house mice or rats, so-called “culture followers”) have been inadvertently favored in the past by human settlements and have unnaturally high populations. These of course attract predation by local cats and is what people see in their own back yards and gardens. This is not necessarily representative, however, of cat predation impacts elsewhere (see Section 5 on biodiversity, below). Unfortunately for the cats, analysts tend to forget other anthropogenic factors influencing prey populations, e.g., habitat loss due to expansion of housing areas, elimination of rodent prey by various means, and replacement of endemic plant species with exotics, among other things (14); factors that are more difficult to account for than is the singling out of a “scapegoat” such as the local predatory activities of cats.

## 3. Studies purporting to show the massive effect of cats on wildlife

Coming back to the two studies receiving the most international media attention mentioned at the outset of this review, both implied or were interpreted by others to indicate alarming predation of house cats on prey populations.

Churcher and Lawton (2) investigated predation by ca. 70 cats in one *English village* over a 1-year period, based on *prey brought home* (535 mammals, 297 birds, 258 remains unidentifiable). Of the identified prey, 17% were wood mice, 16% house sparrows and 14% bank voles. They calculated an average of 14 prey items were brought home per cat per year but presumably many more prey were consumed in the field. *Prey types varied with position within the village*: Core cats brought in more birds than did cats on the edge of the village. The authors estimated that *30% of the sparrow deaths in the village were due to cats*, but stated that the village sparrow population was much higher than the average in other British villages. Although the authors were cautious in their interpretation, the media took off with alarming extrapolations of these very limited data across all of the UK.

Loss et al. (3), in a study of importance to the Lynn et al. (1) *Conservation Biology* article inspiring this essay, conducted a thorough literature review of free-ranging domestic cat predation on birds and mammals in the U.S.A. They acknowledged that cats have contributed to multiple wildlife extinctions on islands [as Fitzgerald (7) previously concluded] but stated that the magnitude of mortality in mainland areas was largely speculative. Their systematic literature review to quantitatively estimate mortality caused by cats concluded that they kill 1.4 to 3.7 billion birds and 6.9 to 20.7 billion mammals in the U.S.A annually. They also stated that un-owned cats (as opposed to owned pets) cause the majority of this mortality and concluded that free-ranging cats are likely to be the single greatest source of anthropogenic mortality for U.S. birds and mammals. However, most of the field studies in their literature review and in their data extrapolation have not taken the above-mentioned known *facts* about cat predatory behavior into account, although their own calculations based just on prey carried home are correct. Among the various studies they considered many lacked a correction for prey eaten or left when away from home, different methods of gut analysis, no control for habitat where the data were collected (suburban, city, farmland), or other causes of prey decimation (e.g., habitat destruction). The media had a field day nonetheless and was quite biased against cats with reports e.g., in *USA Today* [(15), CBS News (16), or BBC World Service (17)].

But the most serious criticism of all such studies is that none of them even mentions a rough estimate of the total population size of a prey species (supposedly being threatened by cat predation) or of the yearly reproduction and replacement of lost individuals. What good does it do to headline that “Cats kill up to 3.7 billion birds annually” if the estimated total population of birds in the USA is *at a minimum* 10 billion *pairs* breeding every year and that as many as *20 billion* are in the country during the fall migratory season [US Fish and Wildlife Service (18), cited January 19, 2011]? Free-ranging cats might be taking about 10–15% of the population of birds annually, but that is not exceptional for a normal predator-prey relationship and is insufficient to eliminate a prey species. Further, estimates of the owned and non-owned free-ranging cat populations are just that–rough estimates.

To date there has been only one “long-term” (3-year) field study by ornithologists to determine the effect of cat predation on a songbird species: Black redstarts (*Phoenicurus ochruros*) which were thought to be particularly vulnerable to nest predators (cats) in a high cat-density area (19). The authors measured yearly production and mortality attributable to cats. Predation by cats caused 33% of egg fatalities, 20% of nestling deaths, ca. 10% of fledgling fatalities and ca. 3% of adult losses. Their conclusion: Predation by cats indeed reduced the productivity of this population by 12% (from 1.20 to 1.06) but did not convert it into a “sink” population. The rate of population increase was sufficient to retain “source” population status. The current author suggests that this might be an exception and highly recommends more such studies before “judgment” is passed on the local cat population.

## 4. Methods to reduce predation by cats

While one can agree or disagree with the necessity to find ways to reduce predation by house cats allowed outdoors, it should be mentioned that a number of studies have considered the effectiveness of methods to do this. Quite often wearing collars with small bells is recommended as a deterrent to successful cat predation, especially on birds. But the results of studies are mixed: Both Barratt (20) and Morgan et al. (21) found that rates of predation by belled cats were not significantly reduced. However, Gordon et al. (22) found reductions of 50% for bird and 61% for rodent predation for belled cats in a 6 weeks on/6 weeks off trial.

Calver et al. (23) showed that wearing a bib to interfere with the cat's ability to pounce greatly reduced predation on birds and was somewhat less successful in reducing predation on reptiles, amphibians and mammals.

However, there was no control to assess how the bib affected the cat's welfare, e.g., to climb a tree when chased by a dog. Such a bib probably reduced the cat's welfare in such cases. More recently Willson et al. (24) reported results of field tests with a two-inch wide brightly colored band mounted over a quick release-collar: Cats wearing this colorful band killed 19 times fewer birds than un-collared cats in the spring trial and 3.3 times fewer birds in the Fall. Small mammal predation was decreased by one half in the Fall.

Finally, the most recent study on methods to reduce cat predation was non-invasive: Cecchetti et al. (25) found that households feeding a high meat protein, grain-free food to their cats, and households where 5–10 min of daily object play with the cats was introduced, recorded decreases of 36 and 25% respectively, in numbers of animals captured and brought home by cats, relative to controls and the pre-treatment period. But again, we have the problem of prey carried home, as mentioned above.

## 5. Effects on biodiversity

There is widespread agreement that biodiversity is important (to our survival) and on the decline (26, 27). Lay conservationists have time and again argued that free-ranging cat predation is reducing biodiversity by eliminating prey species. While this is certainly true for small oceanic islands, Fitzgerald (7) and with the addition of even more field studies (28) have countered that there is simply no evidence that free-ranging cats on the continents are the main cause of species disappearance (and biodiversity reduction) since there is usually a suite of predators utilizing the same prey species and other causes can be cited. Further, the “biodiversity” that most lay conservationists refer to (and see disappearing locally, also with cat predation) is not the only or most important meaning of the word. There are three levels of biodiversity: alpha-, beta-, and gamma biodiversity. *Alpha* diversity is measured very locally in individual habitats; *beta* diversity is a measure of the heterogeneity between habitats while *gamma* diversity (or biodiversity) is the overall species diversity of a range of habitats or communities within a larger region [Oxford Reference for “gamma diversity in ecology,” accessed June 7, (29)]. What we see locally (e.g., in suburbs or villages) is not necessarily representative of what is happening in a wider geographic area. A particular (prey) species may be eliminated locally but thriving in another area or habitat. Beta and gamma diversity are what count (30, 31).

## 6. Free-ranging cats as hosts of zoonotic disease

Lynn et al. (1) also criticize the overgeneralizations and misinterpretation by Loss and Marra (32) and Marra and Santella (33) about the dangers of free-ranging domestic cats transferring zoonotic diseases, and for painting cats as a “looming public health crisis.” This is precisely the type of fear-generating generalization that members of the EU's CALLISTO project on companion animals and zoonoses have cautioned against and have said is unfounded (34).

## 7. Concluding remark

The author does not deny that free-ranging cats affect wildlife populations and it is important that field researchers continue to monitor their effect. But future studies need to take into account what is known about cat predatory behavior, estimates of total prey population size, and interpret the data without prejudice. It remains to be seen whether the media consider and publish reports of less dramatic findings.

## Author's note

This is the English language original, translated by B. Ract-Madoux into French at the publisher's cost for the publication by Dennis C. Turner entitled: “Le chat de compagnie ayant accès à l'extérieur et la faune sauvage: comment surestimer et mal interpréter les données de terrain”. In: Bedossa, Th.; Jeannin, S. (eds.) *Comportement et bien-être du chat*. Dijon: Educagri éditions (2021). Permission granted by Marie GUIOT, head of Educagri éditions.

## Author contributions

The author confirms being the sole contributor of this work and has approved it for publication.
